# External Use of Chloroform in Labor

**Published:** 1884-12

**Authors:** A. Svanberg

**Affiliations:** Sweden


### SELECTIONS.

---

### EXTERNAL USE OF CHLOROFORM IN LABOR. *By* A. SVANBERG, M.D., *Sweden.*

(*Translated by C. W. Johnson, Chicago.*)

Having found it advantageous, during the past few years, in severe cases of labor, where rigidity of the whole organ, or only the internal os, has caused an obstacle to delivery, or prevented some necessary operation or another, to use chloroform externally on the abdomen, and as I have never seen it mentioned in the literature within my reach, I consider I ought to make this method of using chloroform known to the profession. Physicians, to whom I have mentioned this method, have not known of its ever having been so used. For the country practitioner, who so often must perform his obstetrical operations without any assistant other than a midwife of limited knowledge, especially in administering chloroform, it must be a great benefit to be able to avoid using chloroform by inhalation to complete anæsthesia.

During the last few years of my practice I have succeeded in turning, several hours after the rupture of the bag of water, and also in removing retained placenta, without being forced to use chloroform by inhalation, but am convinced that in severe cases it may be necessary to use both methods. I have used it by mixing with sweet oil, equal parts, or chloroform two parts to oil one part, with which I have soaked a piece of flannel, applying it between the symphysis and navel; then, by light strokes over the cloth, make sure it was close to the skin. In severe cases (after five minutes), I soak the cloth the second time, or pour it directly on to the cloth from bottle, where it lies, and by a few gentle strokes the oil is evenly distributed and none is allowed to run down on the vulva. One can thus use the chloroform without oil, but the odor interferes more with the operator, and it takes much more chloroform.

After from five to ten minutes I have always found the rigid and contracted uterus so much lessened that manipulations necessary to be done could be proceeded with.

The first time I used this method was in the spring of 1877. I saw the case with my friend Dr. O. Bloomgren. The placenta was retained for several hours, and patient was very weak from loss of blood. She was put under chloroform for half an hour without being able to remove placenta, on account of rigid contractions. Her condition was now very critical, and we feared to continue chloroform, but resolved to try it externally over the uterus, until her condition was such that it could again be given by inhalation. A flannel soaked in chloroform and oil was applied over the abdomen, and in a short time the placenta came away of itself.

In December, same year, I, with three other doctors, was called to a labor, a primapara, rachitic, and with small pelvis transverse presentation, with arm protruding. The uterus was firmly contracted around the fœtus, and it was impossible to pass the hand into uterus, in view of turning. She was completely anæsthetized, and continued for more than an hour without result. A warm bath was given, then again chloroform, but all in vain. At last I proposed to try chloroform externally, and in about fifteen minutes we proceeded with the turning.

Since that day I have never used chloroform by inhalation for rigid contractions of the uterus.

November 22, 1879, I was called by a doctor to deliver a woman, where he had failed after a trial of from six to seven hours. The pelvis was small, with a breech presentation, and internal os was rigid. Before we proceed to administer chloroform, I wished to try it externally, and this with the result that in twenty minutes after its application could bring down both feet, after which the labor went on without any unusual trouble.

In the winter of 1880, I was called about four miles away, where a woman, four hours before, had been delivered of child, but the midwife could not deliver the placenta. The patient was very weak from loss of blood. The placenta laid entirely within the uterus, the fundus reaching nearly to the navel, and internal os was so rigidly contracted that I could not follow the cord to its origin. After ten minutes application of the chloroform externally, the rigidity disappeared and my hand introduced; placenta was delivered along with a large amount of blood clots. The hemorrhage ceased immediately after the removal of the placenta, and the uterus contracted normally. Made rapid recovery.

On the 15th of July, this year, I was called into the country to see a multipara; the bag of water had ruptured more than seven hours before my arrival, the presentation was transverse, the uterus tightly grasping the fœtus. After applying the chloroform externally, she was delivered of a live child in less than one-half hour. The placenta was spontaneously delivered in ten minutes after the completion of labor. No loss of blood.—*From Eira-Tidskrift for Helso-och Sjukvard.*

